# Perforating collagenosis

**DOI:** 10.1002/ski2.319

**Published:** 2023-12-06

**Authors:** Kazumasa Oya, Aki Saito

**Affiliations:** ^1^ Department of Dermatology Institute of Medicine University of Tsukuba Tsukuba Japan; ^2^ Kashiwa Ekimae Clinic Kashiwa Japan

## Abstract

We show a case of a 69‐year‐old man with perforating collagenosis, which is a rare dermatosis commonly associated with diabetes mellitus. Papules and plaques with keratotic plugs are distinctive clinical characteristic of perforating collagenosis. Representative clinical images in our article can enhance the understanding of key concepts of perforating collagenous.

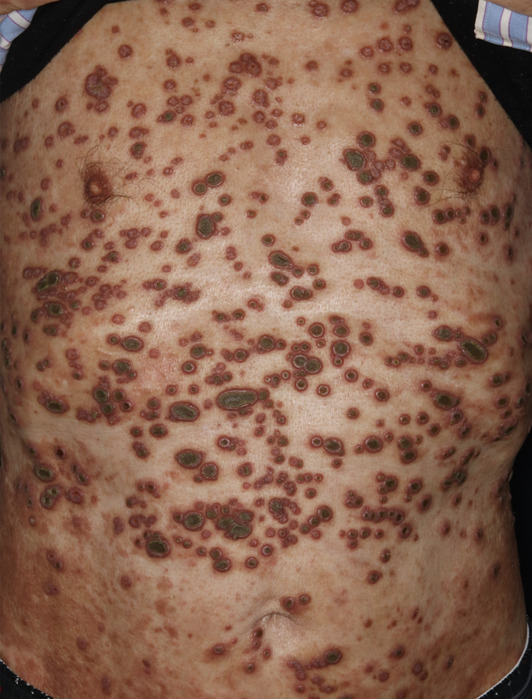

A 69‐year‐old man with diabetes mellitus presented to the dermatology hospital with a generalised rash persisting for 10 weeks. He had neither a history of trauma nor renal failure. Physical examination revealed multiple umbilical plaques on his trunk (Figure 1a, b), and reddish pruritic nodules with partial central ulcerative umbilication in his extremities (Figure 1c), leading to the diagnosis of perforating collagenosis. Perforating collagenosis is characterised by papules with keratotic plugs and pathological findings of the trans elimination of collagen bundles, which is commonly associated with diabetes mellitus.^1^ Clobetasol propionate and diaphenylsulfone, 25 mg twice daily, and bepotastine 10 mg once daily, improved lesions within 2 months. No relapse was observed during the follow‐up period of 2 years with the use of bepotastine.

**FIGURE 1:**
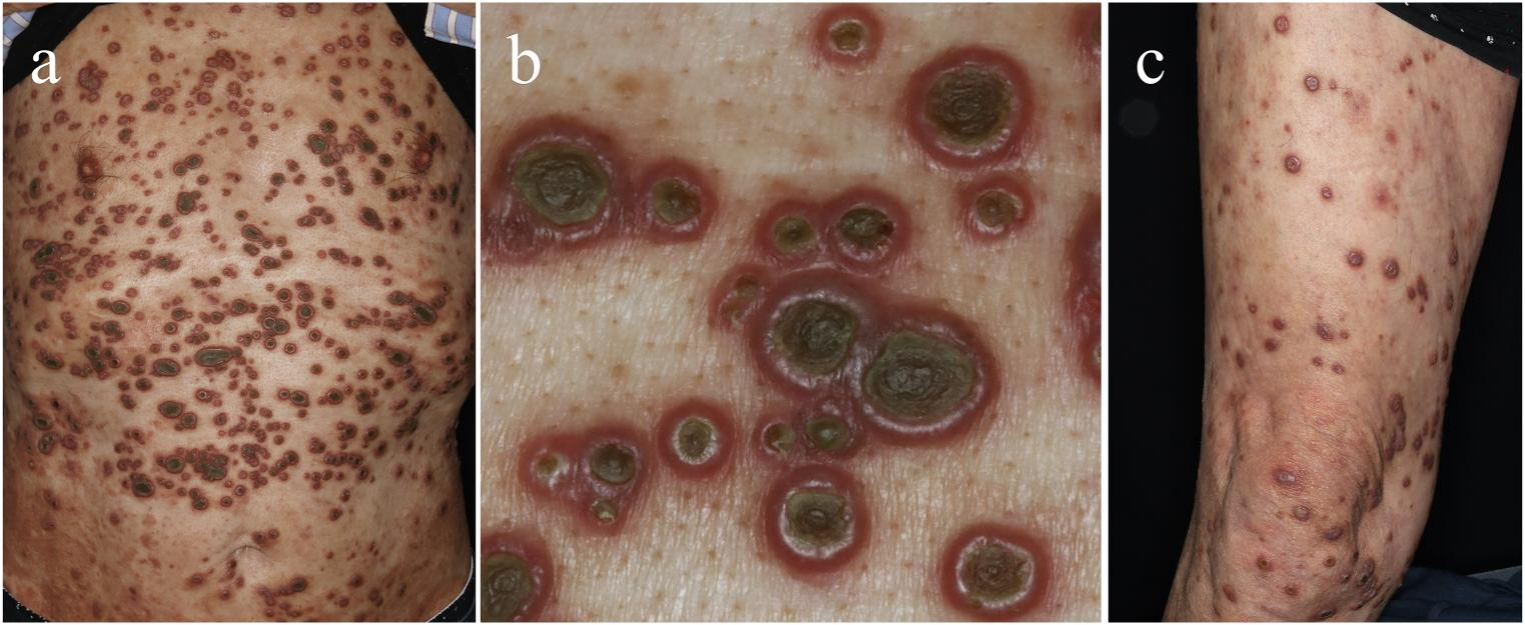
(a‐c) At presentation, multiple umbilical plaques are visible on his trunk (a, b; a closer view) along with reddish pruritic nodules with partial central ulcerative umbilication in his lower extremities (c).

## AUTHOR CONTRIBUTIONS

**Kazumasa Oya**: Conceptualisation (equal); Data curation (equal); Resources (equal); Supervision (equal); Validation (equal); Writing – original draft (equal); Writing – review & editing (equal). **Aki Saito**: Conceptualisation (equal); Supervision (equal); Validation (equal); Writing – original draft (equal); Writing – review & editing (equal).

## CONFLICT OF INTEREST STATEMENT

None to declare.

## ETHICS STATEMENT

Not applicable.

## Data Availability

The data that support the findings of this study are available from the corresponding author upon reasonable request.
